# Machine learning classification techniques for smart agriculture: a review of disease detection and crop recommendation

**DOI:** 10.3389/frai.2026.1878006

**Published:** 2026-09-30

**Authors:** M. Umamaheswari, Kavitha D

**Affiliations:** School of Computer Science and Engineering, Vellore Institute of Technology, Chennai, India

**Keywords:** classification models, convolutional neural networks, internet of things (IoT), machine learning, precision agriculture, random forest, smart agriculture, support vector machine

## Abstract

The adoption of Internet of Things (IoT) and Machine Learning (ML) in agriculture presents a revolutionary step toward efficient agricultural practices using data to address global issues surrounding food security. This review conducted a systematic search of the Scopus, IEEE Xplore, ScienceDirect, and SpringerLink databases for articles published between January 2023 and January 2026. It used keyword combinations like “IoT and machine learning and agriculture”, “crop recommendation”, “plant disease detection”, and “precision farming”. From the initial pool of articles found, 30 articles met the criteria for empirical methodology, quantitative performance reporting, and clear dataset descriptions. These were chosen for detailed analysis. It was discovered that the ensemble, as well as the traditional machine learning methods, such as Random Forest, Support Vector Machine (SVM), and Decision Trees, produce highly accurate predictions of the crop yields with an accuracy of 99.9%. Meanwhile, Convolutional Neural Networks (CNN) and ResNet-based deep learning models detect diseases with an accuracy above 98% while Gated Recurrent Units (GRU) and Long-Short Term Memory (LSTM) time-series models provide forecasts of weather conditions with an accuracy ranging from 94 to 98%, due to the complex nature of the environment dynamics. The application of IoT systems for precision agriculture produced a significant improvement in terms of resource utilization, resulting in 60–96% of water savings, 25% increased yield, and 36–38% reduced herbicide use. Although these advancements have been made, wider application is still hindered by high costs of implementation, lack of technological skills among farmers, data privacy concerns, issues of scalability within different agricultural settings, and consistent internet connection in rural areas. The current paper evaluates the use of IoT and ML in the agriculture sector from diverse data sources and studies to reveal the immense benefits offered by the two systems in enhancing farming activities.

## 1 Introduction

The agricultural sector finds itself at a point in time where conventional methods of cultivation will have to evolve to cope with the growing demands of the population, estimated at nine billion people worldwide in the year 2050. With additional pressures arising from climate change, dwindling resources, and ecological degradation, there is an urgent need for the agricultural industry to make a complete transition toward smarter and more sustainable agricultural processes. The advent of such technologies as Internet of Things (IoT) and Machine Learning (ML) has made this possible. Thus, the modern era of agriculture has come to be known as Smart Agriculture or Agriculture 4.0, utilizing the IoT-powered monitoring of environmental parameters, soil quality, plant condition, and weather trends, followed by the use of ML algorithms to derive meaningful intelligence for the purpose of precision agriculture. Recent advancements have confirmed the applicability of these technologies in a variety of uses within agriculture such as crop yield forecasting, disease detection, weather predictions, irrigation scheduling, and crop suggestions, but major hurdles remain that inhibit adoption, especially in developing nations due to their costly nature, lack of technical knowledge, insufficient infrastructure, and data privacy issues. Under such circumstances, this study provides an in-depth analysis of 30 articles discussing the use of Internet of Things (IoT) and machine learning (ML) in agriculture in various geographical locations, including India, Somalia, and others, and focusing on staple crops like rice, wheat, sugarcane, cotton, potatoes, and vegetables, with the goal of reviewing existing literature on methodology, evaluation criteria, datasets, benefits, drawbacks, and difficulties in smart farming technology, among others.

While many earlier reviews have looked at IoT or ML in agriculture separately, most of them either focus on one specific area, like disease detection, or give a general overview of technology without a detailed comparison of model performance across different tasks. Few reviews examine crop yield prediction, disease detection, weather forecasting, and crop recommendation within the same organized framework, and even fewer compare traditional ML, deep learning, and ensemble methods side by side using the same performance criteria. This review fills that gap by combining 30 recent studies across these four areas, providing a clear comparison of methods, datasets, and performance metrics. It also identifies common technological and practical challenges to real-world use.

## 2 Background and theoretical foundations

### 2.1 Evolution of smart agriculture

The agricultural sector has gone through a series of evolutionary stages, ranging from the traditional and manual form of farming (Agriculture 1.0) to mechanized farming (Agriculture 2.0) and precision farming (Agriculture 3.0) to the latest form of farming, i.e., Agriculture 4.0. This latest form of farming has been described as the integration and implementation of cyber-physical technologies, IoT devices, artificial intelligence, and big data analytics to form intelligent, autonomous, and efficient farming ecosystems.

Agriculture 4.0 has been described as a comprehensive and holistic approach to farming, where different technologies integrate to address the challenges facing the agricultural sector. This has been attributed to the necessity to enhance productivity while conserving the environment, to effectively utilize resources, and to provide food for the ever-increasing population. This transformation in the agricultural sector is, therefore, significant in the sense that the sector consumes approximately 70% of the total freshwater usage globally and has been affected by climatic change (Alzubi and Galyna, 2023; Priya and Anitha, 2025).

### 2.2 IoT in agriculture

Internet of Things technology in the field of agriculture involves the implementation of various sensors, actuators, and communication devices in the form of a network for real-time monitoring and control of agricultural processes. Internet of Things technology has three major layers: perception layer, network layer, and application layer (Selvam et al., 2024). Internet of Things sensors used in the field of agriculture include soil moisture sensors, temperature and humidity sensors (DHT11, DHT22), pH sensors, light intensity sensors, and camera sensors for visual perception (Nik et al., 2023). Internet of Things sensors in the field of agriculture measure parameters like soil moisture content, nutrient content (nitrogen, phosphorus, potassium), temperature, humidity, rainfall, and crop health (Abdullahi et al., 2024). Communication protocols used in Internet of Things technology in the field of agriculture include Wi-Fi, LoRaWAN, Zigbee, Bluetooth, and 5G technology. Internet of Things technology has various communication protocols for different requirements, like the range of communication, power consumption, rate of data transmission, and cost (Omer et al., 2024). Recent advancements in 5G technology have shown better connectivity with low latency, high bandwidth, and capability for handling a large number of Internet of Things devices for real-time monitoring and control of agricultural processes (Selvam et al., 2024).

### 2.3 Machine learning paradigms

Machine learning algorithms allow agricultural systems to learn from the data provided, identify patterns, and predict the outputs without being explicitly programmed. The machine learning paradigms used in smart agricultural systems can be broadly classified into three types: supervised learning, unsupervised learning, and deep learning paradigms.

#### 2.3.1 Supervised learning

The machine learning algorithms used in smart agricultural systems fall under the supervised learning paradigm. The commonly used algorithms include support vector machine (SVM), random forest (RF), decision tree (DT), k-nearest neighbors (KNN), logistic regression (LR), etc. These machine learning algorithms can be used for classification problems such as crop recommendation and disease prediction or regression problems such as yield estimation.

#### 2.3.2 Deep learning

Convolutional Neural Networks (CNNs) have been widely used in smart agricultural systems for image processing-based agricultural applications such as disease prediction and crop classification. Recurrent Neural Networks (RNNs), LSTM, GRU, and their variants have been used in weather prediction and yield estimation. ResNet50, XLNet, and custom CNN models have been used in smart agricultural systems with improved performance.

#### 2.3.3 Ensemble methods

Ensemble methods for classification involve combining several base classifiers to improve classification accuracy. Ensemble methods, such as stacking, bagging, and boosting, were shown to significantly improve classification performance over individual classifiers for crop recommendation and yield prediction.

### 2.4 Integration framework

The integration of IoT and ML in smart agriculture is based on a systematic framework for data acquisition, data pre-processing, model training, model deployment, and decision support systems. IoT technology is used for continuous data acquisition on environmental and crop-related parameters. This data is then sent to cloud or edge computing for data storage and processing. ML models are then trained on past data to identify relationships between input parameters and target variables (Martin et al., 2024; Jiang et al., 2025). After model training, these models are deployed for real-time predictions based on input data received from IoT sensors. This enables farmers to make precise decisions based on spatial and temporal variability in data, thereby maximizing productivity while minimizing resource use. This entire framework enables automation in smart agriculture, where farmers can turn on sprinklers or receive alerts on potential threats to their crops (Akilan and Baalamurugan, 2024; Muninarayanappa and Ranjan, 2023).

The choice between cloud-based and edge-based execution of ML models involves a trade-off between computational capacity and latency/connectivity constraints. In the reviewed studies, most traditional ML and ensemble methods (Random Forest, SVM, Decision Tree) were trained and executed on cloud servers, taking advantage of greater processing power for handling larger datasets. In contrast, TinyML implementations (e.g., quantized CNN models on ESP32-CAM) were specifically designed for on-device (edge) inference, trading some accuracy (87.5–96% vs. 98-99% for cloud-based CNNs) for independence from internet connectivity—an important consideration in rural areas with unreliable network access.

Deploying AI models on embedded agricultural devices presents several distinct challenges:

- Limited memory and processing power require model compression via quantization or pruning.
- Power constraints, since many devices run on batteries or solar power, favor lightweight architectures over deep, high-parameter models.
- Intermittent connectivity complicates model updates and centralized data aggregation, motivating hybrid edge–cloud or federated approaches.

Hardware heterogeneity (e.g., Arduino Mega vs. ESP32-CAM vs. Raspberry Pi) complicates standardized deployment pipelines across different farm setups.

## 3 Methodology

### 3.1 Literature search strategy

This review adheres to a structured systematic methodology to ensure transparency and reproducibility.

#### 3.1.1 Databases searched

The literature search was done in the databases of Scopus, IEEE Xplore, ScienceDirect, SpringerLink, and Google Scholar due to their broad coverage in engineering, technologies, and agricultural sciences.

#### 3.1.2 Publication period

The review concentrated on the literature published during the period of January 2023–January 2026 which is the latest period showing the latest developments in the area of IoT sensors and deep learning architectures.

#### 3.1.3 Keywords and search strategy

A comprehensive search strategy was employed based on the Boolean combination (AND OR) of the following keywords: “Internet of Things (IoT)”, “Smart Agriculture”/"Smart Farming”, “Machine Learning (ML)”, “Precision Agriculture”, “Crop Yield Prediction”, “Disease Detection in Plants”, “Deep Learning in Agriculture”, “UAV in Smart Farming”.

#### 3.1.4 Inclusion criteria

- Papers discussing the application of IoT and ML in agriculture.
- Quantitative data on performance.
- Peer-reviewed journals/conference papers/peer-reviewed review papers.
- Publications made recently (2023–2026).

#### 3.1.5 Exclusion criteria

- Articles older than 2023.
- Papers without detailed methodology/experimental results.
- Non-peer-reviewed materials or only theoretical papers.
- Publications not written in English.

#### 3.1.6 Duplicate removal process

First, the search results underwent several stages of cleaning. The duplicates in the dataset were detected and deleted using reference management software. Subsequently, the title and abstract screenings were carried out to remove any unnecessary articles. In the case of the residual data, the missing values in numeric data sets were addressed through mean imputation or deletion based on the amount of missing values.

PRISMA flow diagram
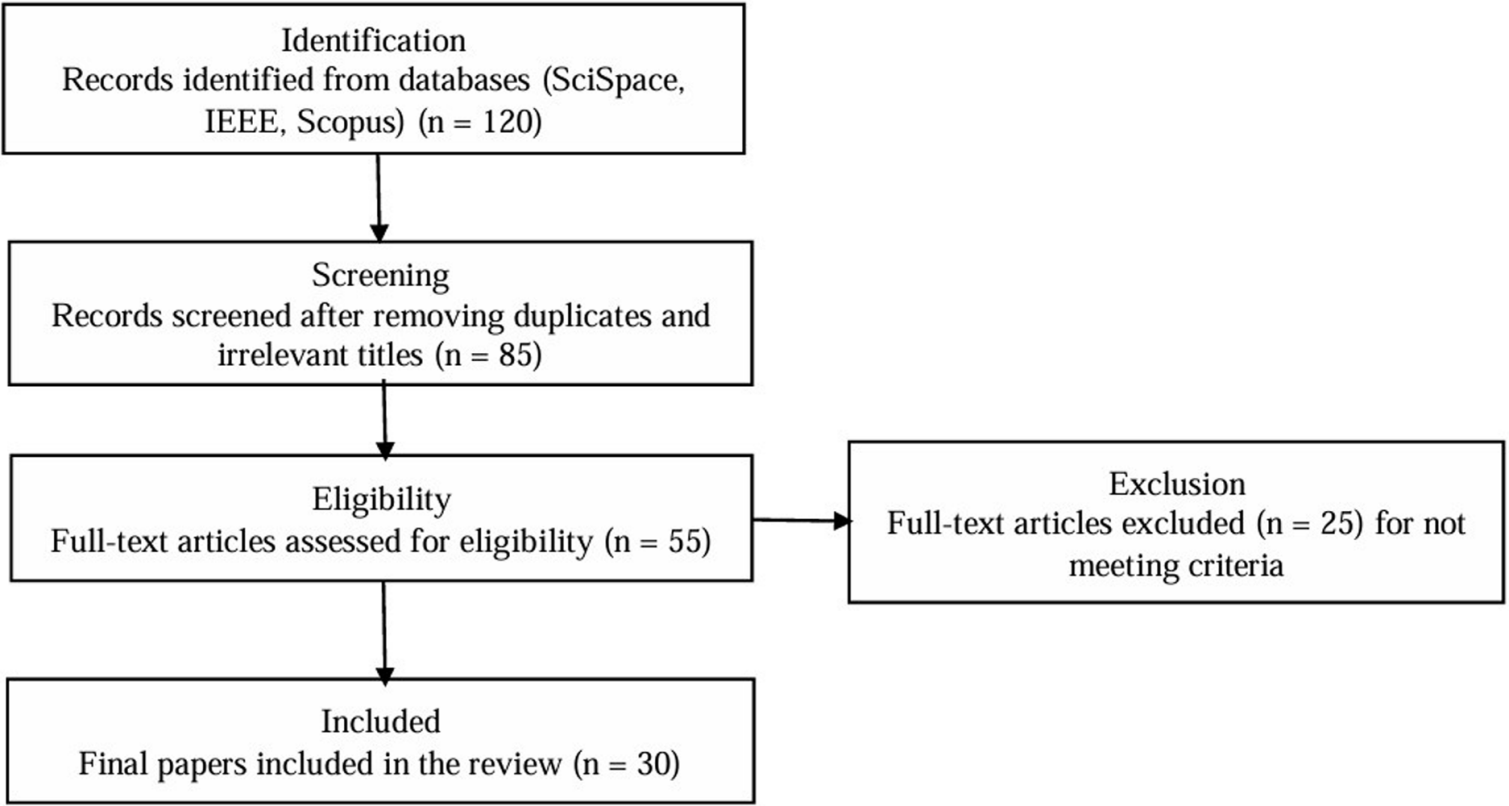


### 3.2 Data collection and preprocessing techniques

Data preprocessing is a significant step in the implementation of smart agriculture. Data preprocessing ensures the quality and suitability of the data for training the ML model. Several methods have been used in the research papers reviewed for data preprocessing. These methods include the following:

#### 3.2.1 Data cleaning

Data cleaning, where missing values and duplicate values are removed, is a basic step in data preprocessing. Missing values and duplicate values in the dataset have to be handled. For instance, missing values in a dataset containing numerical values can be replaced by the mean value if the missing values in the dataset are few (Akilan and Baalamurugan, 2024).

#### 3.2.2 Normalization and scaling

Min-max normalization, where the values in the dataset are normalized to a range [0, 1], has been used in several research papers to ensure uniform scaling of the features in the dataset. This ensures that the features in the dataset contribute equally to the training process (Akilan and Baalamurugan, 2024; Syed, 2024; Abdullahi et al., 2024). Standard scaling or z-score normalization has also been used in several research papers to ensure uniform scaling. Standard scaling ensures that the features in the dataset have a standard deviation of 1 and a mean value of 0.

#### 3.2.3 Feature engineering

This involves the construction of new features based on existing ones. This is done by finding trends in temperature variation, rainfall distribution, etc. Also, time-series data can be converted into weekly or monthly averages. Correlation analysis is performed using Pearson Correlation Coefficients. This helps in finding the most relevant features (Syed, 2024; Abdullahi et al., 2024).

#### 3.2.4 Image preprocessing

In image processing, the following operations can be performed on images:

- Resizing images of different sizes to fixed sizes like (224, 224), (320, 240), (80, 60) pixels.
- Normalizing pixel values by dividing by 255, so the pixel values are between 0 and 1.
- Removing noise from images using Adaptive Gaussian Filters.
- Enhancing image contrast by using Dynamic Histogram Equalization (DHE).

Using data augmentation techniques like flipping, rotating, zooming, and shifting images.

#### 3.2.5 Time series preprocessing

Time-series data can be handled by removing seasonality, trends, and constructing lag features.

Figure 1 depicts the data preprocessing workflow utilized in the preparation of raw agricultural data for training machine learning algorithms. This involves several processes, including data input, data cleaning, normalization and scaling, feature engineering, image data augmentation, dimensionality reduction, and splitting the dataset into training, validation, and testing datasets. At the data input stage, data are gathered and incorporated from various sources such as sensors and images. The next process is data cleaning, in which data noise is eliminated, missing data are eliminated, duplicates are taken care of, and outliers are identified to enhance data quality. Normalization and scaling processes such as Min-Max normalization and standard scaling are applied to transform features so that they fall within a similar range.

**Figure 1:**
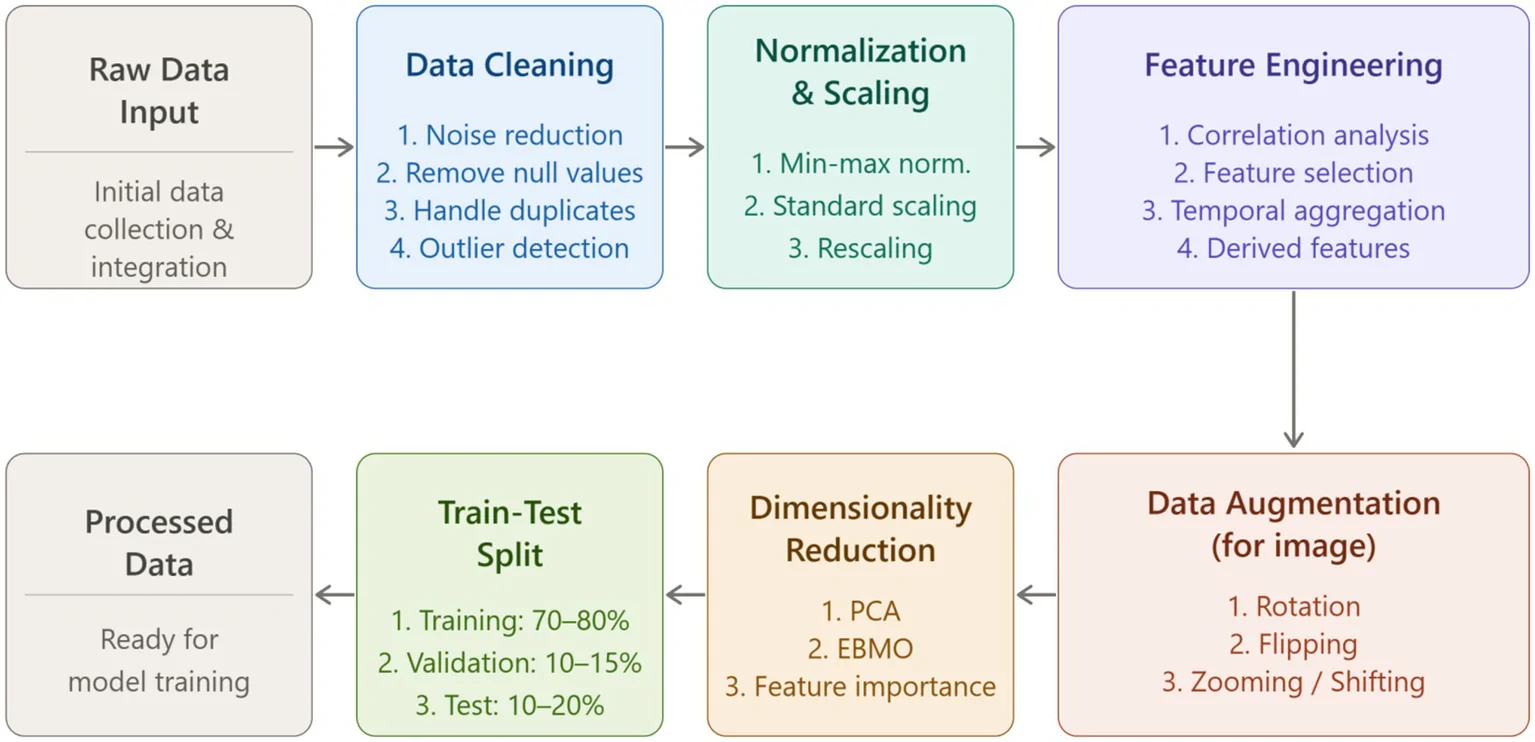
Data pre-processing pipeline.

Feature engineering involves the identification of features by carrying out correlation analyses, feature selections, and generation of derived features. Image data augmentation processes like rotations, flips, zooms, and shifting operations are carried out in image datasets. Dimensionality reduction processes such as PCA, among others, are used at this stage to eliminate unnecessary features.

### 3.3 Machine learning models and architectures

The Smart IoT Agriculture System uses a hierarchical structure, which makes it easy for the farm processes to be monitored and automated. In Figure 2, the bottom most layer is the IoT layer, where sensors like moisture, temperature, humidity, and cameras collect real-time information from the farm fields. This information is transferred using the communication layer using WiFi and LoRaWAN technologies into the cloud platform or edge gateway.

**Figure 2:**
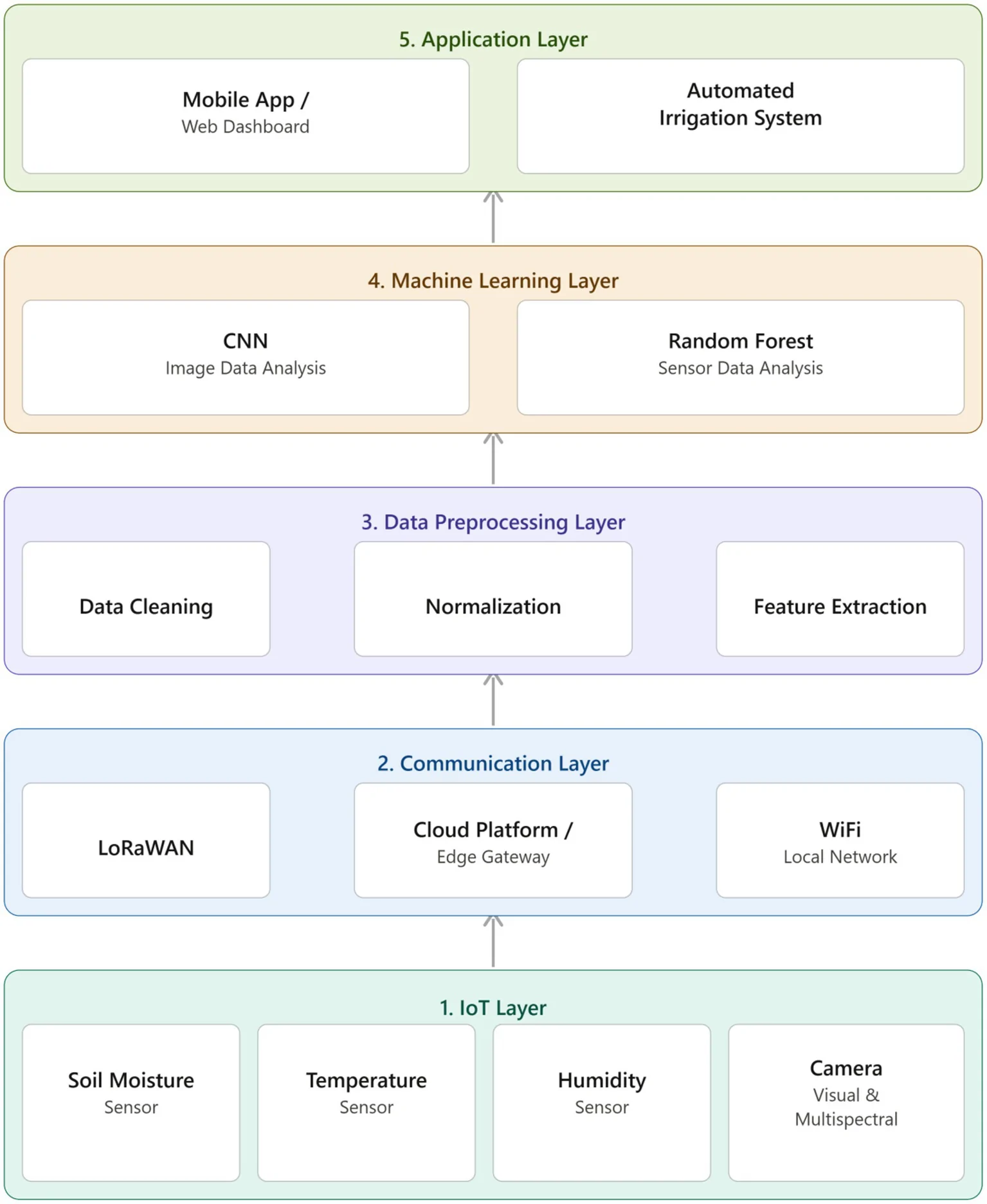
Layered architecture.

The next layer up is the data pre-processing layer, where the information collected is cleaned and normalized in preparation for analysis through feature extraction. At the machine learning layer, the data analysis is done through the use of techniques such as CNN and Random Forests when analyzing image and sensor data, respectively, to aid in disease detection and prediction of irrigation time among other functions. Finally, at the application layer, the information is provided via applications or web dashboards and irrigation automation is performed by mobile applications. These architectural designs reflect the strategic implementation of IoT sensors, preprocessing, machine learning models, and decision-making systems which are integral to the contemporary approach to smart farming (Donskoy et al., 2025; Martin et al., 2024).

#### 3.3.1 Traditional machine learning models

*Support vector machines (SVM)*: SVM models are used for classification in crop recommendation and disease detection (Padmavathi et al., 2024). When coupled with the latest feature extraction technique, i.e., XLNet, the accuracy of crop yield prediction using the SVM model is exceptional. For instance, the accuracy achieved by the proposed model in rice yield prediction was 98.565%, and in cotton yield prediction, the accuracy was 98.986% (Martin et al., 2024).

*Random forest (RF)*: RF is a popular machine learning technique used for crop classification and yield prediction. RF attains a high accuracy of up to 99.7% in crop recommendation systems using a combination of decision trees and the concept of overfitting.

*Decision trees (DT)*: DT provides interpretability and transparency, which makes it appropriate for decision support systems in agriculture. Research has achieved 99.2% accuracy in crop recommendation in Somali agriculture.

*K-nearest neighbors (KNN)*: KNN has been applied in classification problems using similarity-based approaches. Although KNN has demonstrated satisfactory performance, it has relatively lower performance compared to ensemble methods. The accuracy obtained by KNN was 97.2–97.4%.

*Logistic regression (LR)*: Logistic Regression has been applied as a baseline classifier in several studies and has obtained an accuracy of 96.7%.

#### 3.3.2 Deep learning architectures

*Convolutional neural networks (CNN)*: CNNs are the most popular choice for image-based agricultural applications. Various CNN architectures have been employed:

*Custom CNN architectures*: Researchers have developed custom CNNs with several convolutional layers, maximum pooling layers, dropout layers, and dense layers. A six-layer CNN architecture was developed to achieve an accuracy rate of 99% in potato disease detection (Patil et al., 2025).

*ResNet50*: This is an architecture based on residual networks, and it is employed to achieve high accuracy in predicting diseases based on images of crops. An accuracy rate of 98% was achieved with high precision and recall rates.

*Hybrid models based on CNNs*: A CNN-Decision Tree (CNN-DT) model has shown promising results in crop variety classification.

*Gated recurrent units (GRU)*: GRUs are used in weather forecasting. The model attained 94% accuracy in predicting the temperature and moisture content of the soil.

*Long short-term memory (LSTM)*: The LSTM model and its variants, such as HALSTM—Hybrid Attention-based Long Short-Term Memory, are used in the prediction of the smart farming sector.

*XLNet*: The XLNet model is used in the feature extraction process of the crop yield prediction system. The XLNet model, when integrated with the SVM model, attains the best results with an increase in accuracy by 2.563% compared to the conventional model.

#### 3.3.3 Ensemble learning approaches

Ensemble methods involve combining several classifiers to improve the accuracy of prediction results. The two-level ensemble approach involves employing Logistic Regression, CART, SVM, and KNN as level-0 classifiers, followed by employing Random Forest as the level-1 meta-classifier to achieve 99.7% accuracy in crop prediction (Syed, 2024).

#### 3.3.4 Optimization techniques

*Dimensionality reduction*: Enhanced Butterfly Moth Optimization (EBMO) is utilized to reduce the dimensionality of features while retaining important information, thus reducing the complexity of agricultural datasets.

*Model quantization*: For deployment on devices with limited resources, like the ESP32 microcontroller, quantization is done to convert the model to an 8-bit integer format while still retaining acceptable accuracy levels.

*Explainable AI (XAI)*: SHAP (SHapley Additive exPlanations) and LIME (Local Interpretable Model-agnostic Explanations) techniques are used to ensure transparency in AI-based agricultural decisions. Of the 30 studies reviewed, only Martin et al., 2024 explicitly used XAI methods (SHAP and LIME) with an XLNet + SVM yield prediction model. Most of the deep learning studies on disease detection and crop recommendations did not include any interpretability analysis. Grad-CAM, a technique used to show which areas of an input image most affect a CNN’s classification, was missing from all the disease-detection studies reviewed. This is surprising given its importance for helping farmers and agronomists trust and verify automated diagnoses (Verma et al., 2025).

#### 3.3.5 Emerging AI techniques

While most of the reviewed studies focus on traditional supervised learning, CNN, and ensemble methods, a few new techniques are starting to impact smart agriculture research, though they are not well represented in the current literature sample.

*Vision transformers (ViTs)* process images as sequences of patches, using self-attention instead of convolutional filters. They have demonstrated strong results in image classification tasks. None of the 30 studies examined used ViT-based architectures for disease detection or crop classification, highlighting a gap compared to the broader computer vision literature, where ViTs are increasingly matching or outperforming CNNs.

*Foundation models* are large pre-trained models that can be adapted to various tasks through fine-tuning or prompting. These models are gaining popularity in agriculture for tasks like crop identification and satellite image analysis. However, only lightweight LLM-based approaches, such as Farm-LightSeek, were present in the reviewed sample, which focuses on edge-based agricultural data analytics.

*Federated learning* allows model training across multiple farms without the need to centralize raw data, which addresses the data privacy concerns raised in this review. None of the reviewed studies utilized federated learning, even though it is clearly relevant to the data privacy and ownership issues discussed in Section 7.3.

*Edge AI* appears primarily in the reviewed literature through TinyML quantized CNN deployed on ESP32-CAM hardware, achieving accuracy rates between 87.5 and 96% depending on image resolution.

*Multimodal learning* was explored in a small number of studies, such as those by Garg et al. (2025) and Singha Roy and Kukreja (2025). These studies combined image data with sensor and environmental data using dual-branch architectures: CNN for images and MLP for tabular sensor data, achieving accuracy rates between 95 and 98%. This suggests that multimodal fusion is a promising but still emerging area.

### 3.4 IoT infrastructure and sensor networks

#### 3.4.1 Hardware components

*Microcontrollers*: They are based on Arduino Mega, Raspberry Pi 3 B+, and ESP32-CAM boards, etc. ESP32-CAM contains two 32-bit Xtensa LX6 processors, 4 MB PSRAM, and support for external MicroSD cards. Hence, it is best suited for edge computing and AI applications (Donskoy et al., 2025).

*Sensors*: Agricultural IoT infrastructure consists of several sensors such as (see Table 1):

- Soil moisture sensor for irrigation management. DHT11/DHT22 temperature and humidity sensor.
- PH sensor for measuring the acidity levels in the soil.
- NPK sensor for measuring nutrient levels in the soil.
- Light intensity sensor for measuring the rate of photosynthesis.
- Camera sensor (OV2640) for visual inspection.

**Table 1:** Common agricultural sensors used in smart farming.

Sensor type	Parameter measured	Typical range	Accuracy	Operating voltage	Application
Soil moisture sensor	Soil moisture	0–100%	±3–5%	3.3–5 V	Irrigation management
DHT11	Temperature, humidity	0–50 °C	±2 °C	3.3–5 V	Environmental monitoring
DHT22	Temperature, humidity	−40–80 °C	±0.5 °C	3.3–5 V	Environmental monitoring
pH sensor	Soil pH	0–14	±0.1 pH	5 V	Soil analysis
NPK sensor	N, P, K levels	Sensor dependent	±5–10%	5–12 V	Fertility assessment
Light sensor (BH1750)	Light intensity	1–65,535 lux	High sensitivity	3.3–5 V	Photosynthesis monitoring
OV2640 camera	Image data	2 MP	High resolution	3.3 V	Disease detection

*Communication module*: IoT devices in agriculture use wireless communication modules such as Wi-Fi to transmit sensor information to the server. Advanced IoT devices also utilize 5G technology.

#### 3.4.2 IoT network architecture

IoT-based systems used in agriculture use a three-tier architecture, which includes:

*Perception layer*: The sensors deployed at each location within the farm environment gather information. Each sensor comprises sensors, a microcontroller, power supply (batteries or solar energy), and a communication component.

*Network layer*: Data received by the sensors is aggregated by gateway devices (Raspberry Pi) and transmitted to the cloud and fog computing layers (Akilan and Baalamurugan, 2024). Data transmission occurs via Wi-Fi, LoRaWAN, and 5G technology.

*Application layer*: ML models run on cloud servers or edge computing nodes, and data can be visualized and controlled through applications.

#### 3.4.3 Data storage and processing

*Cloud computing*: The cloud offers scalable storage and computational power for training complex ML models as well as storing large amounts of past data. For applications requiring low latency, *Edge computing* processes data on gateway nodes or microcontroller. This reduces data transmission requirements and allows for autonomous. *Fog computing*: the intermediate processing between edge nodes and cloud servers is provided by fog nodes.

## 4 Related work analysis

This chapter will discuss the review of related works on smart agriculture through IoT and ML in a detailed manner. This review will be presented through three comparative tables based on temporal, methodology, and characteristic comparisons (see Tables 2 and 3).

**Table 2:** Comparative analysis of methodologies, performance and limitations.

Author	Year	Methodology used	Performance/Accuracy	Limitation
Raj and Prahadeeswaran	2025	PRISMA systematic review; Analysis of 79 papers on AI, UAV, IoT, ML in agriculture	Random forest: 99.7%, SVR: 99.9% *R*^2^, Disease detection: >90%, Fire detection: 98.77%	High implementation costs, Limited technical expertise and Unreliable internet access; Data security concerns
Alzubi and Galyna	2023	AI and IoT integration for sustainable farming; Comprehensive technology review	Demonstrated potential for 20% yield improvement	Large data management and compatibility issues; Depend on stable internet connectivity and technical expertise
Akilan and Baalamurugan	2024	GRU for weather forecasting; ResNet50 (CNN) for disease detection; IoT sensors (Arduino Mega, DHT11, soil moisture)	Weather forecasting (GRU): 94%, Disease detection (ResNet50): 98%	High implementation cost due to IoT sensors and infrastructure; Limited datasets and environmental parameters may affect generalization.
Sharafat et al.	2025	IoT and ML integration for smart farming using Sensor networks and data analytics. Used ML and DL models to recommend suitable crops.	Random Forest-95.8%, Gradient Boosting-95.5%, and a stacked SVC ensemble −95.9%. TabNet achieved 92% accuracy	Uses a region-specific dataset which may limit generalization; Limited dataset size and crop diversity may affect scalability in other agricultural environments.
Awasthi et al.	2024	IoT-based smart farming with ML algorithms; Real-time monitoring and prediction	Improved decision-making and resource management	Manual NPK input may introduce human errors; High initial implementation cost and technical complexity for farmers.
Garg et al.	2025	MLP works on sensor data, CNN works on images for feature extraction. The extracted features are used by the final MLP for classification into three classes of plant diseases.	Hybrid model accuracy is 95%. The model exhibits high precision, recall, and *F*1-score values of up to 0.98, 0.96, and 0.97, respectively.	Implementation and maintenance costs of IoT devices, cloud infrastructure, and computational resources may limit large-scale field deployment.
Selvam et al.	2024	Custom CNN (6 conv layers, 6 maxpooling, dropout, flatten, 2 dense); 5G IoT integration; Potato leaf disease dataset	CNN accuracy: 99%, Precision: 98%, Recall: 98%, *F*1-score: 98%	High initial setup cost for IoT sensors, 5G infrastructure, and cloud systems. Limited dataset that only potato leaf images it may reduce model generalization for different crops.
Syed	2024	Two-level ensemble: LR, CART, SVM, KNN (Level-0); Random Forest (Level-1); Pearson correlation for feature selection	Ensemble: 99.7% accuracy, 99.68% precision, 99.66% recall; CART: 98.63%, SVM: 97.7%	The system depends heavily on accurate sensor data; any missing or noisy data can reduce prediction reliability. High accuracy may not generalize well to different region.
Bouni et al.	2024	The data undergoes pre-processing and is analyzed by ML algorithms such as random forest, decision tree, and LightGBM.	Random Forest achieve 99.31% and other models LightGBM achieved 98.90% and decision tree achieved 98.48%	Farmers may lack technical knowledge and training to use the system. Poor internet connectivity can affect real-time data collection and predictions.
Muninarayanappa and Ranjan	2023	IoT sensors data are gathered and structured through the KNN classification technique. The structured data are then analyzed in Hadoop MapReduce framework.	The accuracy is very high at 98.9%, and the model enhances performance by reducing both CPU time and memory consumption.	Hadoop distributed infrastructure which increases implementation cost; Performance may vary with different datasets and requires further evaluation to reduce storage and processing cost.
Abdullahi et al.	2024	Decision tree, KNN, random forest; IoT sensors for soil NPK, pH, temperature, humidity, rainfall	Decision tree: 99.2%, Random forest: 99.0%, KNN: 97.2%	Dataset was collected only from one region, so the model may not work well in other locations. Only a few machine learning algorithms were tested.
Khaliq et al.	2025	TTL model is used for irrigation advice, SwiFT model for crop recommendation, and TabNet with XAI for soil analysis and fertilizer recommendation.	99.13% accuracy for irrigation, 98.75% for crop recommendation, and 99.3% for fertilizer prediction, along with strong soil analysis *R*^2^ = 98.7%.	Models may not generalize well across different regions, soils, and climates without retraining. High cost, maintenance issues, and need for technical knowledge can limit adoption by small-scale farmers.
Donskoy et al.	2025	TinyML CNN on ESP32-CAM; Two CNN architectures tested; Model quantization to 8-bit integer	CNN Architecture 2 (quantized): 96.15% (320 × 240), 92.86% (160 × 120), 87.50% (80 × 60)	Low power devices, cannot handle big or complex models. Accuracy may drop with small images or compressed models. Needs good quality data to work well in real conditions.
Roy and Kukreja	2025	A multimodal deep learning model combines CNN-based image features and MLP-based environmental data. The system uses a dual-output architecture with multi-task learning to simultaneously detect 10 disease classes and 3 severity levels.	The model achieved 97.9% accuracy and 97.6% *F*1-score for disease classification, outperforming models like ResNet and EfficientNet.	Needs good quality data to work well Requires high computing power Can be affected by noise and changing environment conditions
Shifna and Kadarkarai	2025	Hybrid Artificial Long Short-Term Memory (HALSTM) model is used for prediction and classification, along with the Fused Red Piranha Grey Wolf Optimization (FRPGW) algorithm for feature selection.	The proposed model achieves high performance with 94% accuracy, 95% precision, 90% recall, and 87% *F*1-score, outperforming existing methods.	It is trained mainly on Indian data, so it may not work well in other regions or for all crops. It also uses data only up to 2020 and does not include some important factors like pests and soil biology and its performance may vary depending on data quality and computational resources.

**Table 3:** Summary of datasets used in smart agriculture research.

Author and year	Dataset name/Type	Source/Origin	Dataset characteristics	Data modalities
Alzubi and Galyna (2023)	IoT-based smart agriculture data (sensor and environmental data)	Data collected from IoT devices, sensors, drones, and smart farming platforms	Real-time agricultural data including soil moisture, temperature, humidity, crop health, weather conditions, and irrigation information	Sensor data, environmental data, weather data, drone/UAV images, and farm management data
Akilan and Baalamurugan (2024)	Agricultural sensor data, soil nutrient dataset, crop production dataset, crop leaf image dataset	Ministry of Agriculture and Farmers Welfare (India), Agricultural sensor network, Soil nutrient database, Crop disease image dataset	Includes crop area, season, temperature, soil moisture, soil nutrients, and 3,600 RGB crop leaf images (healthy and diseased) used for weather forecasting and disease detection	Multimodal data: Sensor data (temperature, humidity, soil moisture), Soil nutrient data (N, P, K, pH, EC), Agricultural production data, and RGB crop leaf images for disease classification
Sharafat et al. (2025)	Private crop prediction dataset (tabular dataset) collected from multiple survey and analysis	Soil Resource Development Institute (SRDI) and Bangladesh Agri-Meteorological Information Service (BAMIS), Bangladesh	Contains 3,300 samples covering 22 crop types with 8 soil and environmental features. The dataset is balanced with about 150 samples per crop and collected from Habiganj district, Bangladesh.	Soil and weather data: Nitrogen (N), Phosphorus (P), Potassium (K), soil pH, temperature, humidity, soil moisture, and rainfall
Awasthi et al. (2024)	IoT smart farming dataset (sensor + image data)	Collected from field sensors and camera system connected to Raspberry Pi	Includes environmental parameters such as temperature, humidity, soil moisture and manually entered NPK values; image data used for detecting animals, pests, and crop conditions.	Multimodal data: numerical sensor data (temperature, humidity, soil moisture, NPK values) and image data for object detection using YOLO
Garg et al. (2025)	Jamun plant disease dataset (sensor + image dataset)	Collected at DA-IICT campus, Gandhinagar, India using IoT sensors and smartphone	6,227 sensor data samples collected every 30 min for 6 months and 4,195 leaf images (2,389 anthracnose, 658 leaf spot, 1,148 healthy). Images resized to 224 × 224 and sensor data normalized.	Multimodal dataset combining numerical sensor data (ambient temperature, soil temperature, humidity, soil moisture, leaf wetness duration) and leaf images for disease detection.
Selvam et al. (2024)	Potato leaf disease dataset (image dataset)	Kaggle—potato leaf disease gallery	Contains 2,152 potato leaf images divided into 3 classes: Early Blight (1000), Late Blight (1000), Healthy (152). Images are resized to 256 × 256. Dataset split 80% training and 20% testing.	Image data (RGB leaf images) used for plant disease classification using CNN.
Syed (2024)	Crop recommendation dataset (open-source)	Kaggle/IoT sensor-based data	Contains 7 features and 22 crop categories; used for training and testing ensemble ML models; includes environmental and soil parameters.	Numerical sensor data: Temperature, Humidity, pH, Rainfall, Nitrogen (N), Phosphorus (P), Potassium (K)
Bouni et al. (2024)	Crop Recommendation and yield prediction dataset (secondary agricultural dataset)	Online data repository	Around 2,200 records with 8 attributes including Nitrogen (N), Phosphorus (P), Potassium (K), temperature, humidity, pH, rainfall, and crop label used for ML model training and testing.	Numerical sensor data such as soil nutrients (NPK), environmental parameters (temperature, humidity), soil pH, rainfall, and crop class labels.
Muninarayanappa and Ranjan (2023)	Crop monitoring sensor dataset (gas sensors for home activity monitoring dataset used for agriculture analysis)	UCI machine learning repository	919,438 data records with 11 attributes such as id, time, R1–R8 sensor readings, temperature, and humidity; multi-dimensional and unstructured sensor data used for crop/environment monitoring	IoT sensor data including temperature, humidity, and gas sensor readings
Abdullahi et al. (2024)	IoT-based crop recommendation dataset	Collected from farms in Afgooye, Balcad, and Jowhar districts, Somalia using IoT sensors	Dataset contains 1,000 samples with 10 crop types (Banana, Coconut, Kidney beans, Maize, Mango, Lemon, Papaya, Rice, Watermelon, Pumpkin). Data collected from May 2022–April 2023 and split 75% training/25% testing.	Soil and environmental parameters: Nitrogen, Phosphorus, Potassium (NPK), pH, temperature, humidity, rainfall collected through IoT sensors.
Khaliq et al. (2025)	Multi-source agricultural dataset (tabular + sensor data)	Public datasets + Simulated IoT sensor data + weather API	Includes soil nutrients (N, P, K), pH, moisture, temperature, humidity, rainfall; cleaned, normalized, encoded; supports multiple tasks (crop, fertilizer, irrigation, soil analysis).	Tabular data, IoT sensor data (soil and environment), weather data (API), historical agricultural data
Donskoy et al. (2025)	Combination of custom + public crop/weed image datasets (image classification)	Self-collected using ESP32-CAM + external datasets (Kaggle, DeepWeeds, GWHD, etc.)	12,272 images, multiple resolutions (32 × 32 to 800 × 600), includes classes like weed, wheat, and plant-free zones, augmented and preprocessed.	RGB images (low to medium resolution), image data for classification
Singha Roy and Kukreja (2025)	Rice leaf disease dataset (multimodal-image + environmental)	Collected from real field conditions (Bolar Kalan, Punjab) + environmental logs	2,265 images, 10 disease classes, 3 severity levels (mild, moderate, severe), balanced distribution, real-world images with noise and variability.	Image data (leaf images) + Environmental data (temperature, humidity, rainfall, soil pH, irrigation, etc.)
ShreneShifna and Kadarkarai (2025)	Agricultural crop yield dataset (time-series data)	Indian states dataset (1997–2020), from Kaggle	Contains 10 features such as crop, state, season, area, rainfall, production, fertilizer, pesticides, and yield; multi-year, multi-crop data.	Numerical data (rainfall, yield, production), Categorical data (crop type, state, season), Time-series data

## 5 Findings and comparative analysis

### 5.1 Crop yield prediction systems

Crop yield prediction forms the core of agricultural planning and decision making. As seen from the reviewed works, considerable progress has been made in terms of prediction precision due to the utilization of IoT sensors and ML models.

*Prediction with XLNet*: The most advanced framework uses XLNet for feature extraction paired with an SVM classifier which allows obtaining outstanding results: accuracy of 98.565% for rice crops, 98.098% for sugarcane, 98.656% for sorghum, 98.354% for Rabi crops, and 98.986% for cotton. The prediction model uses a rich array of information from reliable sources, including meteorological data (NASA POWER), soil data^1^, and crop production data (ICRISAT) for 34 districts of South India spanning from 2001 to 2015. To solve the problem of high computational load due to processing of massive agriculture data, EBMO algorithm for feature selection was used (Martin et al., 2024).

*Ensemble models*: A two-stage model using LR, CART, SVM, KNN as base classifiers and RF as meta-classifier reached 99.7% prediction precision on 22 crop types. In comparison with base classifiers, a better result was obtained by ensembling models: CART—98.63%, SVM—97.7%, KNN—97.4%, LR—96.7% (Syed, 2024).

*Traditional ML algorithms*: Random Forest algorithm always proves to be efficient, scoring 99.7% accuracy in classifying crops based on data from Kaggle. Support Vector Regression showed excellent results in predicting the wheat production rate, providing 99.9% *R*^2^ accuracy for predictions made in 2025. As seen, even traditional ML algorithms, if properly optimized, can provide outstanding accuracy levels on well-prepared datasets (Raj and Prahadeeswaran, 2025; Syed, 2024).

*AI explainability*: Usage of SHAP and LIME explanations as part of the XLNet + SVM method helps clarify how AI-based agricultural decisions are made, overcoming “black-box” limitations of deep learning algorithms. Explainability is key for adoption by farmers since it allows gaining insights into decision-making processes (Martin et al., 2024).

### 5.2 Disease detection and plant health monitoring

Plant diseases detection is one of the important applications where timely diagnosis could help avoid substantial crop damage. Deep learning, especially the use of CNNs, has made great progress in this field (Balafas et al., 2023).

*Custom CNN architecture*: A customized six-layer CNN architecture showed a 99% accuracy in diagnosing diseases in potato leaves, including early blight, late blight, and healthy. The proposed model had outstanding performance with respect to precision (98%), recall (98%), and *F*1 score (98%) compared to existing methods. The combination with 5G IoT technology would allow real-time detection and reaction to the identified disease.

*ResNet50 model:* The implementation of ResNet50, which stands for residual neural networks, was able to achieve 98% accuracy in disease prediction based on crop images, with similar levels of precision, recall, and specificity at 98%. This system uses Adaptive Gaussian Filters and Dynamic Histogram Equalization to improve image quality before processing. It can automate disease management actions like pesticide spraying and notify farmers about any detected diseases via SMS.

*CNN hybrid models*: The hybrid model of a CNN and decision tree model for various Indian French beans showed excellent results for accuracy, precision, recall, and *F*1-score. It is an approach that combines the strength of both the models for disease detection.

*Edge-based AI model for disease detection*: The TinyML based edge machine learning approach using ESP32-CAM microcontroller can perform local disease detection without relying on the cloud platform. The accuracy rate obtained by the quantized CNN model for classification was 87.50% at the resolution of 80 × 60 pixels with classification time between 4–15 s. It classified the images as plant-free area, weeds, and wheat images.

*Comparison with performance measures*: In different studies, the accuracy rates for disease detection were greater than 90%, and many were 95–99% (Raj and Prahadeeswaran, 2025; Akilan and Baalamurugan, 2024; Selvam et al., 2024; Donskoy et al., 2025). The variance in accuracy rates was due to the difference in datasets and resolution used in the experiments.

### 5.3 Weather forecasting and environmental monitoring

Weather forecast is very important for the purposes of crop cultivation, planning irrigation timings, etc. The implementation of Internet of Things sensors in conjunction with machine learning models based on time series has greatly advanced weather forecast abilities.

*GRU based weather forecast:* Gated Recurrent Unit (GRU) models achieved a forecast accuracy of 94% for both temperature and soil moisture content measurements with precision, recall, and *F*1-score equal to 94, 93, and 94%, respectively. The model uses min-max normalization technique during data preparation as well as mean imputation to fill up any missing data. It should be noted that lower forecast accuracy compared to diseases prediction can be explained by the nature of weather changes which are quite stochastic (Akilan and Baalamurugan, 2024).

*Variants of LSTMs*: The use of hybrid attention-based LSTM and feature reduction using Particle swarm optimization and genetic algorithm with wrapper approach can improve the ability of the forecast in intelligent farming practices (ShreneShifna and Kadarkarai, 2025).

*IoT sensor network*: IoT sensor network involves the use of wireless sensor network that uses microcontrollers such as Arduino Mega and temperature and humidity sensors including the DHT11 sensor and soil moisture sensors as well as light intensity sensors to gather real-time data on environment conditions. Raspberry Pi 3 B+ acts as the gatekeeper of the network as it collects and sends collected data from different sensor networks to the cloud-based servers for analysis (Akilan and Baalamurugan, 2024).

*Automated responses*: On account of weather predictions as well as collected data from the IoT sensor network, automated responses include activating water pumps for irrigation, adjusting greenhouses and issuing alerts to farmers about possible adverse weather conditions.

### 5.4 Irrigation management and water conservation

Water shortage has become a significant issue within the agricultural industry, necessitating effective irrigation management practices. IoT-ML technologies have shown impressive results in terms of water conservation, coupled with increased agricultural productivity.

*Water conservation accomplishments*: Smart irrigation systems, based on IoT, recorded water conservation of up to 60% while increasing crop productivity by 25%. Precision irrigation using UAVs resulted in water conservation rates of 92–96%. ML-driven irrigation systems recorded up to 60% in water conservation due to improved irrigation scheduling using soil moisture information, weather forecasts, and crop water demands.

*Soil moisture sensing*: Real-time monitoring of soil moisture content facilitates efficient irrigation planning. This technology uses a threshold-based control mechanism where irrigation operations start when the moisture level drops below crop-specific thresholds and are suspended once adequate levels are attained.

*Prediction of irrigation needs*: Applications of AI have proven 98.4% accurate in predicting irrigation needs by utilizing weather forecasts, changes in soil moisture, and plant growth stages. Predictive ability enables the farmer to schedule irrigation requirements in advance.

*Automated systems*: IoT-based automated water pumps use sensor readings and machine learning predictions without any human supervision, thus providing optimal irrigation. Automation of such systems provides an added advantage especially in large farms, where human monitoring is difficult.

### 5.5 Crop recommendation systems

Crop recommendation is done to recommend farmers the most appropriate crop that suits the environmental factors such as weather conditions, type of soil used, etc. (Abdullahi et al., 2024).

*Performance of decision trees*: Decision Tree algorithms performed at 99.2% accuracy in crop recommendation for Somali agriculture. The precision, recall, and *F*1-score recorded were 99.0%. Decision trees have high levels of interpretability, which make it easy to explain the decisions made.

*Performance of random forest*: Random Forest performed at 99.0% accuracy in crop recommendation. Random forest has the ability to handle feature interaction well due to its nature of being an ensemble of models.

*Performance of K-nearest neighbor algorithm*: K-Nearest Neighbor algorithm performed at 97.2% accuracy in crop recommendation. It had a precision, recall, and *F*1-score of 96.0%.

*Feature importance*: It was found through the Pearson correlation test that soil pH, NPK, temperature, humidity, and rainfall are the main factors affecting the crop recommendation process. An understanding of feature importance leads to efficient methods of soil improvement and environmental management (Ahmed et al., 2024).

*Regional adaptation*: In the study conducted in Somalia, customized IoT data from the regions of Afgooye, Balcad, and Jowhar were used, comprising 10 crops locally grown within these districts, with each sample consisting of 100 samples of each crop. This helps ensure that recommendations are made in line with the regional conditions and climate changes. Nevertheless, the use of a region-specific database poses challenges in its generalization to other parts of the globe.

*Real-time crop recommendation*: The inclusion of IoT into the system makes it possible to give real-time recommendations based on the prevailing soil and climatic conditions, providing the farmer with timely information regarding planting.

### 5.6 Comparative analysis of machine learning models in smart agriculture

To provide a clearer analytical perspective, the performance of machine learning and deep learning models reported in the reviewed studies was compared across major smart agriculture applications. Table 4 summarizes the reported performance metrics of representative models used for crop recommendation, disease detection, weather forecasting, and IoT-enabled monitoring systems.

**Table 4:** Comparative analysis of machine learning models.

Application	Algorithm	Accuracy	Precision/Recall/*F*1	Computational complexity	Interpretability	Real-time suitability
Crop recommendation	Random forest ensemble	99.7%	99.68/99.66/—	Moderate (multiple trees)	Moderate	Yes (lightweight)
Disease detection	CNN (custom, 6-layer)	99.0%	98/98/98	High (training), low (inference)	Low (black-box)	Yes (edge-deployable)
Disease detection	ResNet50	98.0%	98/98/98	High	Low	Limited (needs GPU)
Weather forecasting	GRU-CNN	94.0%	94/93/94	Moderate–High	Low	Yes
Edge AI disease	TinyML CNN (quantized)	87.5–96.0%	—	Low (post-quantization)	Low	Yes (designed for edge)

Based on the results of the studied articles, it is possible to note that the ensemble method is better in solving crop recommendation problems due to the capability to use multiple decision trees in order to avoid overfitting. The deep learning approaches like Convolutional Neural Network (CNN) and models based on ResNet architecture demonstrate excellent precision in detecting diseases using images as the source data due to automatic features extraction (Sangeetha et al., 2024).

It can also be noted that the performance of the models largely depends on the properties of the used dataset such as its size, the ratio of classes, the quality of features and geographic diversity. However, even though there are multiple papers with accuracy rates above 95%, the possibility of fair performance comparison is limited due to dissimilarities in experiments (see Figure 3).

**Figure 3:**
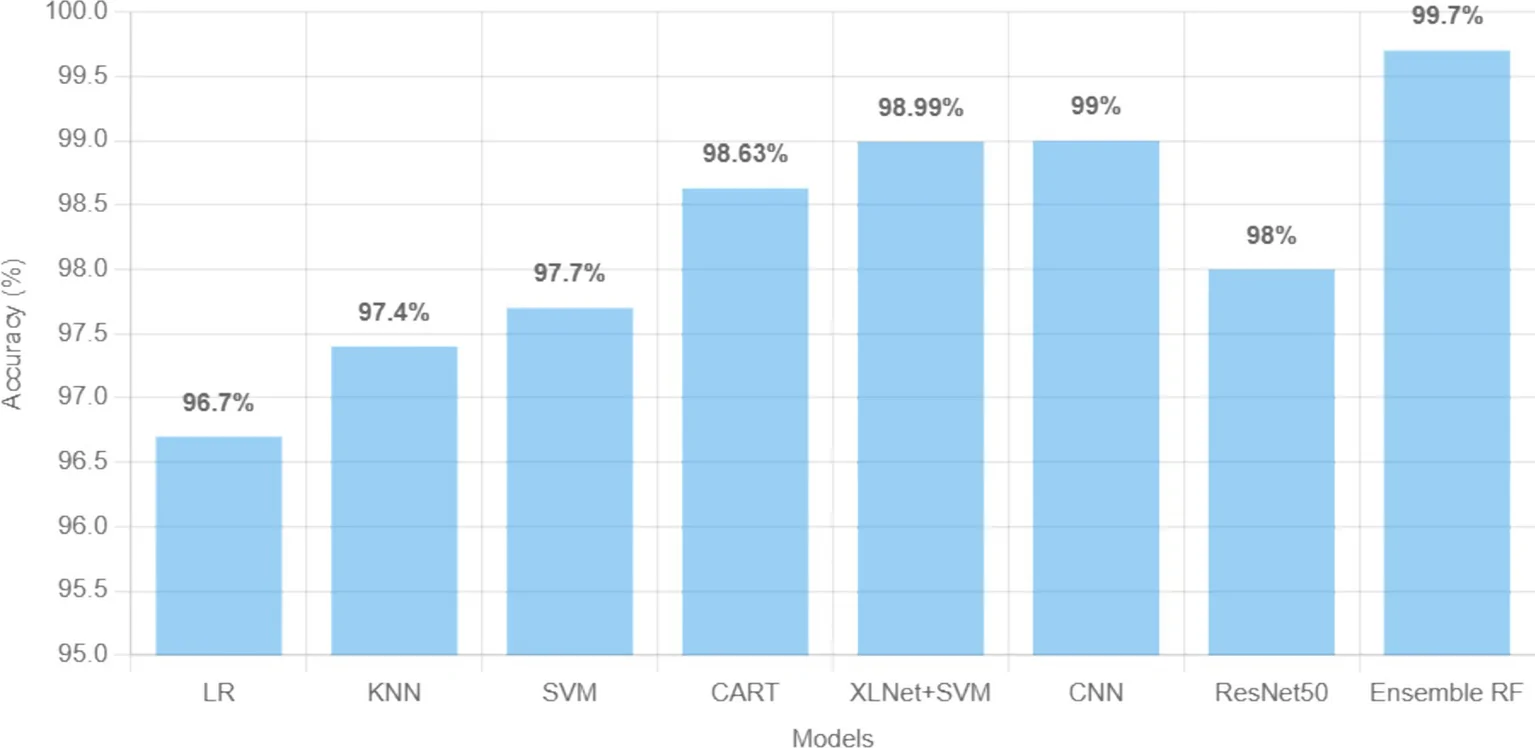
Comparative analysis of machine learning models.

### 5.7 Model interpretability in smart agriculture

The aspect of model interpretability has not been adequately explored in the literature reviewed. Although ensemble methods such as Random Forest and Decision Trees provide inherent interpretability via feature importance rankings and by showing the decision paths, deep learning models (CNN, ResNet50, GRU, LSTM) work mostly as black boxes. This is a major drawback for agricultural decision-support systems, since farmer trust and regulatory accountability rely on being able to understand the reasons why the model recommends a certain crop, flags a disease, or predicts a yield. The only one of the studies reviewed incorporated formal XAI techniques (SHAP, LIME) into a high-performing deep learning pipeline. Greater use of interpretability techniques—such as SHAP, LIME, and Grad-CAM for image-based models—is required in order to close the gap between model performance and their practical, trustworthy application in the field.

## 6 Data analysis

Diversity and representativeness of data sets play crucial roles in ensuring the efficiency of ML-based applications in smart farming. This part discusses the data sets used in different studies presented above.

### 6.1 Dataset sources and types

#### 6.1.1 Public datasets

Some researchers work with public datasets, specifically those from Kaggle. The Kaggle dataset named “potato leaf diseases Gallery,” comprising 2,152 images in three categories (early blight, late blight, healthy) is used in disease detection studies. Public datasets provide benefits like standardization, replicability, and lower data acquisition costs, although they may not reflect region-specific situations (Selvam et al., 2024).

#### 6.1.2 Datasets from government and institutions

The best dataset source uses data from several authenticated sources of government data in India such as https://geoportal.natmo.gov.in/ for soil data, https://data.icrisat.org/ for crop production data, and https://power.larc.nasa.gov/ (NASA POWER) for weather data (Martin et al., 2024). Such multi-source data are collected from 34 districts spread across five south Indian states (Tamil Nadu, Andhra Pradesh, Telangana, Karnataka, Kerala) over 15 years (2001–2015) (Martin et al., 2024).

#### 6.1.3 Custom data collection using IoT sensors

Several papers rely on the collection of custom data through IoT sensor deployments. For instance, the Somali agricultural research paper collected data from three districts, Afgooye, Balcad, and Jowhar, through IoT sensors that recorded soil nitrogen content, potassium content, phosphorus content, soil pH, air temperature, humidity, and rainfall between May 2022 to April 2023. Although custom data is relevant to the study context, its collection entails substantial costs (Abdullahi et al., 2024).

#### 6.1.4 Hybrid methods

A few research works use external databases in addition to custom data collected by the researchers (Donskoy et al., 2025).

The above Table 5 represents a description of the main datasets utilized in the studied works related to smart agriculture. The datasets vary depending on their source, size, feature set, modality, and pre-processing methodology. Publicly available databases such as Kaggle, Plant Village, NASA POWER, and ICRISAT have been commonly utilized in research due to their large collections of annotated agricultural data. Pre-processing methods include data cleaning, normalization, feature selection, image augmentation, and feature extraction. Training and testing samples from datasets are usually split into proportions of 75:25 to 80:20 for model validation purposes. The variety of datasets indicates that machine learning approaches can be applied successfully across a wide range of applications in smart agriculture.

**Table 5:** Comparative summary of datasets used in smart agriculture.

Application	Dataset source	No. of instances	Parameters used	Data format	Preprocessing	Train/Test split
Crop yield prediction	NASA POWER, ICRISAT, geoportal soil database	34 districts (2001–2015)	Rainfall, temperature, humidity, soil properties, crop production	Tabular	EBMO feature selection, normalization	Reported in study
Crop recommendation	Kaggle crop recommendation dataset	2,200 records	N, P, K, temperature, humidity, pH, rainfall	CSV	Cleaning, scaling	80:20
Potato disease detection	Kaggle potato disease dataset	2,152 images	RGB leaf images	Image	Resizing, augmentation	80:20
Plant disease detection	PlantVillage dataset	>50,000 images	Plant leaf images	Image	Image enhancement, augmentation	80:20
Jamun crop monitoring	DA-IICT dataset	6,227 sensor records + 4,195 images	Soil moisture, temperature, humidity, images	Multimodal	Normalization, feature extraction	Reported in study
Smart agriculture Somalia	Field-collected IoT dataset	Approximately 1,000 samples	NPK, pH, rainfall, temperature	Tabular	Data cleaning	75:25

### 6.2 Data modalities

#### 6.2.1 Sensors data

The most typical form of the used data modalities is comprised of environmental and soil data gathered using IoT sensors like,

#### 6.2.2 Soil data

Moisture, pH level, nutrients (nitrogen, phosphorus, potassium), soil structure (sand, silt, and clay ratios), organic matter, temperature.

#### 6.2.3 Weather data

Temperature (daily max/min and average), precipitation amounts and patterns, humidity, wind speed, solar radiation.

#### 6.2.4 Crops data

Phenological phases, productivity per hectare, vegetation indices (NDVI, EVI), pests and diseases, harvest periods.

#### 6.2.5 Images data

Images acquired using cameras or drones include the following:

#### 6.2.6 Crop diseases images

Images in RGB format depicting both healthy and diseased crops.

#### 6.2.7 Field monitoring images

Overhead images for weeding and crops categorization.

#### 6.2.8 Image resolution types

Researchers use different resolutions ranging from high (1,600 × 1,200 and 512 × 512) to low (80 × 60) according to their needs.

#### 6.2.9 Time-series data

Sensor data obtained in time-series allows researchers to use data for forecasting purposes:

#### 6.2.10 Weather time series

Historical patterns of weather conditions for forecasting.

#### 6.2.11 Soil moisture time series

Soil moisture data for irrigation management.

#### 6.2.12 Crops growth time series

Information about temporal stages of plants’ growth.

### 6.3 Dataset characteristics and scale

*Time periods covered*: Data sets cover varying time periods:

*Long-term historical data*: 15 years (2001–2015) for full crop yield analysis.

*Seasonal data*: 1 year of seasonal changes (May 2022–April 2023).

*Continuous real-time data*: Continuous data recording for live operation systems.

*Geographical extent*: Extent covers different geographical regions:

*Regional*: 34 districts across five states in India.

*Multi-site local area*: Three districts in Somalia.

*Single-site application*: Single farm applications.

#### 6.3.1 Sample size

*Large-scale sample*: Thousands of samples recorded during different years and locations.

*Medium-scale sample*: 1000–2000 samples collected for different types of crops or diseases.

*Small-scale sample*: Hundreds of samples recorded for research and application purposes.

*Dimensionality of features*: The number of features covered range between 7–10 basic features (temperature, humidity, pH, NPK, rainfall) to multi-dimensional features including dozens of features covering weather, soil, and crops (Martin et al., 2024).

### 6.4 Data quality and preprocessing

*Handling missing data*: Research uses different approaches for handling missing data:

*Mean imputation*: Replace missing numeric values with the average of that feature if there are only a few missing points.

*Complete case analysis*: Discard rows with too many missing points.

*Interpolation*: Estimate missing values from neighboring points in time series data.

*Data augmentation*: In image-based datasets, augmentation techniques create more diverse samples:

*Geometric manipulations*: Rotation (±15°, ±30°), flipping (horizontally/vertically), zooming (±20%), shifts.

*Color manipulations*: Changing brightness, contrast, and saturation.

*Different resolutions*: Train models on images of varying sizes.

*Data normalization*: Uniform scaling guarantees proper contribution of features during model training:

*Min-Max Scaling*: Scales data between [0, 1].

Z-score standardization: Scaling features to zero mean and unit variance.

*Image normalization*: Dividing pixel values by 255 for scaling between [0, 1].

### 6.5 Dataset limitations and challenges

*Region-specific datasets*: Most datasets tend to be region-specific due to data collection from various parts of the world with unique climatic conditions, soil characteristics, and agricultural practices. For instance, the dataset on agriculture in Somalia largely consists of data collected from Balcad district.

*Limited time span*: Datasets that cover a narrow time span might fail to capture long-term climatic changes, extreme weather occurrences, and crop rotation practices. For example, seasonal data sets do not provide inter-annual variations.

*Imbalanced classes*: Some datasets are characterized by an imbalanced distribution of classes, such as the potato disease database with 1,000 cases of early blight, 1,000 cases of late blight, and 152 healthy potatoes.

*Costs of data collection*: Gathering data using custom Internet of Things involves huge expenses for installing sensors, communication infrastructure, power sources, and maintaining the system.

*Data privacy and security*: Agri-data may comprise sensitive information regarding the farm business and performance. Data security and privacy need to be ensured for wide application.

*Sensors failure risk*: IoT sensors may suffer from sensor failure, drifts, and environmental factors that may compromise their readings and hence the quality of data gathered. Effective techniques must be developed to counter such problems.

*Limitations of pH and NPK sensors*: Soil pH and NPK sensors are some of the most popularly used sensing equipment in smart agriculture. Nonetheless, there are various factors that affect the effectiveness of their readings. For instance, soil pH sensors usually have a detection range of 0–14 pH units and have an accuracy of around ±0.1-±0.2 pH units. There are several issues that can affect their readings, including changes in temperature, soil moisture, electrode aging, and calibration problems that require regular recalibration.

In a similar, NPK sensors are used to approximate the amount of Nitrogen, Phosphorus, and Potassium in a specific range provided by the respective manufacturer. The accuracy of these sensors depends on factors such as soil composition, soil salinity, organic matter, moisture level, and many others. NPK sensors also vary in terms of sensitivity, tolerance levels, and precision in measurement. Additionally, any reading made beyond the minimum and maximum detection limits of the sensors leads to unreliable data collection. Such issues may influence the effectiveness of the training phase of machine learning models as well as predictions. Thus, appropriate sensor choice, regular calibration, testing against lab values, and good data processing are critical for collecting valid data.

*Lack of standardization*: Lack of standardized data formats, collection practices, and quality criteria across different studies makes data sharing and generalization difficult. Developing agricultural data standard will help enhance research in the field.

Despite this Challenges, as seen in the literature discussed above, appropriate datasets that are either publicly or institutionally available, or collected through proper processes, can help in developing accurate machine learning algorithms (Martin et al., 2024).

## 7 Discussion

### 7.1 Performance trends and patterns

According to the findings from literature review, there are some evident performance trends associated with smart agriculture algorithms. Firstly, ensemble techniques and Deep Learning structures significantly outperform single classifier architectures, providing above-95% accuracy, even exceeding 99% in some cases. Such performance levels mean that current ML algorithms have practically reached the maximum accuracy level that could be obtained within a given set of data quality parameters. However, performance metrics show noticeable differences depending on application domain. The highest values of accuracy (95–99%) are observed for disease detection and crop recommendation applications because of the clarity of the classification task. In contrast, weather forecasting models demonstrate lower accuracies due to the probabilistic nature of the process (94%) (Akilan and Baalamurugan, 2024). Yield predictions demonstrate wide variation in accuracy (86–99%) dependent on the type of the cultivated crop and other factors. Another noticeable trend includes integrating explainable AI techniques (SHAP, LIME) into highly performing algorithms, solving the interpretability problem and facilitating farmers’ acceptance of black box ML (Martin et al., 2024). The Edge AI applications show that accuracy ranging from 87 to 96% could be attained in devices with restricted resources due to the quantization of the model. This information is important because it helps in developing smart farming technologies in areas with poor connectivity and low computational capabilities (Donskoy et al., 2025).

### 7.2 Technological challenges

Even with commendable performance metrics, there are some remaining technological challenges including:

*Scalability*: Almost all existing work showcases proof of concept on small geographical scales (individual farms or small areas only). Achieving scalability to regional and national level involves overcoming various challenges such as sensor deployment, data transmission, computational infrastructure, and generalization (Selvam et al., 2024).

*Computational needs*: Deep learning-based approaches involving complex architectures such as XLNet need substantial computing power to train the models. This poses a challenge to agriculture-related institutions with limited computing power. Although edge deployment improves the efficiency of inference, training is computationally demanding (Martin et al., 2024).

*Data quality and availability*: High performing algorithms require significant amounts of data that are well labeled. In many agricultural regions, especially those in developing countries, the necessary infrastructure for gathering such data may not be readily available. Also, faulty sensors or environmental interference can affect data quality (Nawaz and Babar, 2025).

*Connectivity and infrastructure*: Internet connectivity is crucial for IoT applications, allowing for data transmission and processing in the cloud environment. Many rural areas face connectivity problems. Although edge computing solves this problem, it complicates the design and maintenance of IoT systems (Donskoy et al., 2025).

*Interoperability and standardization*: Absence of standardized protocols related to the format of collected data and data transfer creates difficulties for integration of elements from different suppliers. Non-standardized IoT infrastructure complicates implementation and raises expenses (Raj and Prahadeeswaran, 2025).

*Model generalization*: Machine learning models trained on data collected in a particular region may fail in other geographical zones. Transfer learning techniques can help overcome this challenge (Martin et al., 2024).

*Energy efficiency*: Energy efficiency is a critical requirement for IoT sensors powered by batteries. Using power-efficient communication protocols and duty cycling allows for more efficient battery use, but alternative approaches such as solar charging should be considered (Selvam et al., 2024).

### 7.3 Practical implementation consideration

*Cost–benefit analysis*: Justification for high costs associated with IoT setup, sensor equipment, communication technologies, and ML development lies in improved yield, reduced resources, and minimized risks. Small farmers face greater cost challenges than others. Possible solutions include shared technologies, subsidies from governments, or cooperation initiatives (Raj and Prahadeeswaran, 2025).

*Technical skills*: Technical competencies in IoT hardware setup, data transfer, analytics, and ML are essential for deploying and monitoring smart farming systems. However, most farmers do not possess such skills, making special training, technical assistance, or user-friendly interfaces essential (Abdullahi et al., 2024).

*User interfaces*: User interface design plays a crucial role in the process of smart farming, particularly in relation to farmers. Application design should be simple, mobile, in local languages, as well as informative with recommended actions highlighted (Badshah et al., 2024).

*Maintenance and repair*: IoT devices need occasional calibration, battery changing, and repair. Setting up maintenance infrastructure and developing supply chains to get spare parts becomes crucial for sustainability purposes (Saranya and Sankaradass, 2025).

*Privacy and data ownership issues*: Data ownership and privacy become serious barriers in farmers’ willingness to share information because there is no guarantee about how and where it will be used. The absence of such policies makes farmers distrustful.

*Government policy incentives and regulations*: Policies by government can foster implementation of IoT technologies via financial incentives and technical support programs. Data and IoT device safety regulations help assure consistent results.

### 7.4 Social economic implications

*Food security*: Smart farming technology can make a considerable impact on improving food security through increased yields, decreased crop loss, and efficient use of resources. The reported 20% increase in yields and 25% increase in productivity may significantly contribute to an increase in food production, especially in areas affected by food insecurity (Saranya and Sankaradass, 2025).

*Environmental sustainability*: Savings in water usage up to 60–96% and decrease in the use of herbicides up to 36–38% show environmental advantages that can be brought about through precision farming. Resource efficiency is essential for sustainable agriculture amidst climatic changes and resource limitations (Alzubi and Galyna, 2023).

*Economic impact*: An increase in productivity, reduction in costs of inputs such as water, fertilizers, and pesticides, and minimal crop losses would result in improved livelihoods for farmers. The high cost involved may cause economic disparity between big farms and small farmers.

*Digital divide*: The deployment of smart agriculture technologies could cause a further disparity between advanced farms and the farms without access to technology, capital, and infrastructure. Overcoming the digital divide will require targeted efforts such as providing subsidies for technologies and offering training in underserved regions (Badshah et al., 2024).

*Labor issues*: The use of technologies for automation of irrigation, disease control, and other agricultural processes decreases labor needs and could lead to unemployment of farm workers. At the same time, new jobs will arise for maintenance of sensors and data analysis. Thus, workforce transition strategies will be needed (Raj and Prahadeeswaran, 2025).

*Access to knowledge*: Data-driven advice and AI-powered recommendations make agricultural knowledge accessible to everyone. Such democratization of information will allow small-scale farmers to use advanced knowledge that was available only to big companies before.

*Resilience to climate change*: Weather forecasts, adaptive irrigation practices, and disease early warning systems contribute to the resilience of the agricultural sector to climate variability and extreme weather events. Resilience in agriculture becomes particularly important under climate change conditions.

### 7.5 Research gaps and common challenges

Synthesizing across the 30 reviewed studies, several recurring gaps and limitations emerge that cut across individual application domains:

#### 7.5.1 Common challenges

- Nearly all studies report performance on region-specific, single-location datasets, limiting generalizability.
- Very few studies validate models across multiple geographic regions or crop types within the same study.
- Comparative evaluation across studies is hindered by inconsistent reporting of metrics (accuracy alone, without precision/recall/*F*1, in several cases) and differing train/test split conventions.

#### 7.5.2 Research gaps

- A near-total absence of federated learning approaches despite clear relevance to the data privacy concerns raised by farmers.
- Minimal use of explainability techniques beyond a single study, despite repeated acknowledgment that interpretability is key to farmer trust.
- Limited exploration of transformer-based architectures (ViTs, attention mechanisms beyond LSTM/GRU variants) for image and time-series tasks.
- Little longitudinal validation — most datasets cover one growing season or a few years, leaving open questions about model robustness under long-term climate variability.

#### 7.5.3 Limitations of existing methods

High-accuracy models (CNN, ResNet50, ensemble methods) are consistently trained and validated on relatively small, curated datasets (hundreds to a few thousand samples), raising the risk of overfitting to specific conditions not representative of large-scale, heterogeneous farm environments.

#### 7.5.4 Future research opportunities

Priority areas include:

- Multi-region, multi-season benchmark datasets to enable fair cross-study comparison.
- Standardized reporting protocols requiring precision/recall/*F*1 and interpretability metrics alongside accuracy.
- Integration of federated learning for privacy-preserving multi-farm model training.
- Systematic evaluation of transformer-based and foundation models against existing CNN/ensemble baselines.
- Longitudinal studies assessing model performance stability across multiple growing seasons and changing climate conditions.

## 8 Future directions and recommendations

The future work directions for smart agriculture include combining a multitude of information from satellite imaging, drones, IoT devices, weather prediction and historical data using state-of-the-art multimodal fusion algorithms for developing highly intelligent farming frameworks. Modern techniques such as transformer architectures and attention mechanisms are quite effective for modeling relations between different heterogeneous data. Transfer learning, domain adaptation, and few-shot learning can enable generalization of models across various crops and locations while minimizing their reliance on huge datasets. In addition, federated learning allows for model training across farms without accessing any raw data, providing an opportunity for preserving the privacy of each user. Causal inference is another interesting area that would enable making decisions based on actual causal dependencies rather than simple correlation analysis. Finally, reinforcement learning could be employed to automate the processes of irrigation, fertilizing, and pest management. Uncertainty estimation, e.g., by using Bayesian approaches, may help estimate how accurate the predictions are. Longitudinal modeling and XAI are also important for sustainable farming.

Technologically and operationally, standardization of data formats, communication protocols, and evaluation metrics will play an indispensable role in realizing interoperability and scalability in smart agriculture solutions. Innovations in edge AI technology, hybrid cloud-edge computing, and 5G networks will make real-time, low-latency decisions possible, even in resource-limited rural settings. Blockchain technology can facilitate secure and transparent data sharing and tracing, and digital twin technology can create virtual models that mimic farm conditions. Government policies that include subsidies, infrastructure improvements, and education programs will be crucial for promoting adoption, particularly among small farmers. Collaboration between the public and private sectors, as well as data platforms, will further drive innovations and knowledge dissemination. Sustainability and ethics must also take precedence in promoting smart agriculture solutions to avoid adverse effects on the environment and promote equality, algorithmic justice, and farmer independence.

## 9 Conclusion

A thorough literature survey of 30 research articles highlights the revolutionary impact of integrating IoT and ML on the agricultural industry. The research findings have revealed that a sophisticated IoT-based sensor network coupled with highly accurate ML algorithms provide outstanding accuracy (98–99.9%) for crop yield prediction, disease detection, weather forecasting, and crop recommendations The technology stack comprises several machine learning frameworks, from traditional approaches such as Support Vector Machines (SVM), Random Forests, and Decision Tree methods to advanced deep learning architectures such as Convolutional Neural Networks (CNN), Recurrent Neural Networks (RNN), Gated Recurrent Units (GRU), and even transformer models such as XLNet. An ensemble learning method consistently outperforms individual classifiers by generating 99.7% accuracy via the intelligent aggregation of base learners. Moreover, the recent development of Explainable AI (XAI) techniques such as SHAP and LIME solves the interpretability problem, thus establishing trust among farmers. The practical applications have shown many advantages such as water savings of 60–96%, crop productivity improvement of 25%, crop yield improvement of 20%, and reducing the usage of herbicides by 36–38%. The resource optimization is vital when dealing with the major global challenges such as hunger, climatic changes, and environmental sustainability. The successful implementation of edge AI on resource-limited devices (ESP32-CAM) shows the possibility of deployment of highly efficient algorithms in the connectivity-limited environment with 87–96% accuracy and efficiency. But there are also significant challenges. Expensive implementation process, insufficient technical background of farmers, poor infrastructure in rural locations, data security issues, and lack of scalability limit the deployment of the technology especially in the developing countries. The existence of the digital divide creates an opportunity for the emergence of technological inequality between well-equipped commercial farms and small farms which lack resources, infrastructure, and sufficient technical skills. Dataset limitations are regional, time-based, imbalanced, and not standardized.

The way ahead would need concerted efforts on many fronts. Research needs to focus on multimodal data fusion, transfer learning, federated learning, causation, and uncertainty quantification for increasing robustness and applicability of models. Technology advancements require prioritizing standardization, edge AI improvement, 5G compatibility, and sensor innovations for reducing costs and improving access. Subsidies, investments in infrastructure, skill development initiatives, PPPs, and policy measures are required to democratize access and benefit equitably. Sustainability and ethics must inform technological innovations, addressing issues related to environmental sustainability, accessibility, algorithmic biases, autonomy of farmers, and sustainability of agriculture in general. Innovations in smart agriculture should complement the expertise of the farmer, integrating data science-based decision-making with conventional agricultural practices. IoT integration with machine learning technology in the agricultural sector not only signifies technological development but rather implies a paradigm shift toward Agriculture 4.0—the era of smart, intelligent farming systems that can tackle issues associated with feeding an ever-growing world population and preserving natural resources despite global warming. There is plenty of scholarly evidence that this transition is not only possible from the technical perspective but is also necessary, providing the desired results to justify further development. While smart agriculture technologies become more advanced and affordable every day, their contribution to the evolution of global food production, the improvement of the lives of farmers and sustainable development in general becomes increasingly evident. However, it remains crucial to implement scientific findings into practice and promote the development of intelligent farming across the board, especially among poor farmers who can benefit most from data-driven technologies.

## Data Availability

Publicly available datasets were analyzed in this study.

## Footnotes

1
https://geoportal.natmo.gov.in/
